# Performance evaluation of the Access HBsAg and Access HBsAg confirmatory assays on the DxI 9000 Access Immunoassay Analyzer

**DOI:** 10.1016/j.plabm.2024.e00390

**Published:** 2024-03-15

**Authors:** Benoit Visseaux, Jérémie Gautier, Françoise Le Boulaire, Catherine Coignard, Claire Vincent, Sandrine Gréaume, Isabelle Voisin, Veronique Lemée, Jean-Christophe Plantier, Yves-Edouard Herpe, Etienne Brochot, Stephanie Bord, Marc Turini, Vanessa Roulet, Juliane Hey

**Affiliations:** a Laboratoire Cerba, Saint-Ouen l’Aumône, France; b Cerba Xpert, Saint-Ouen l’Aumône, France; c Infectology, Specialized CoreLab Department, Eurofins Biomnis, Ivry-Sur-Seine, France; d Biomnis Sample Library Department, Eurofins Biomnis, Ivry-Sur-Seine, France; e PLER Laboratory, Etablissement Français du Sang Hauts-de-France, Normandie, Bois Guillaume, France; f Laboratoire de Virologie, Institut de Biologie Clinique, Hôpital C. Nicole CHU Rouen, Rouen, France; g Centre de Ressources Biologiques Biobanque de Picardie, CHU Amiens Picardie, Amiens, France; h Laboratoire de Virologie, Centre de Biologie Humaine, CHU Amiens Picardie, Amiens, France; i R&D Department, Beckman Coulter, Immunotech, Marseille, France; j Clinical Affairs Department, Beckman Coulter, Immunotech, Marseille, France

**Keywords:** Hepatitis B infection, HBsAg, Chemiluminescence immunoassay, Analytical performances, Clinical performances

## Abstract

**Introduction:**

This study evaluated the clinical and analytical performances of the Access HBsAg and the Access HBsAg Confirmatory assays on the DxI 9000 Access Immunoassay Analyzer (Beckman Coulter, Inc.).

**Materials and methods:**

Diagnostic specificity and sensitivity of the Access HBsAg and Access HBsAg Confirmatory assays were evaluated by comparing the Access assays to the final HBsAg sample status determined using the Architect, PRISM, or Elecsys HBsAg assays, along with Architect or PRISM HBsAg Confirmatory assays. Imprecision, sensitivity on seroconversion panels, analytical sensitivity on WHO, and recognition of HBV variants were also evaluated.

**Results:**

A total of 7534 samples were included in the analysis (6047 blood donors, 1032 hospitalized patients, 455 positive patients’ samples). Access HBsAg assay sensitivity and specificity were at 100.00% (99.19–100.0) and 99.92% (99.82–99.97), respectively. Sensitivity of Access HBsAg Confirmatory assay was 100.00% (99.21–100.0) on the 464 HBsAg positive samples. The use of a high positive algorithm for the Access HBsAg assay, wherein samples with S/CO ≥ 100.00 were considered positive without requiring repeat or confirmatory testing, was successfully evaluated with all 450 specimens with S/CO greater than 100.00 (sensitivity 100.00%; 99.19–100.0). Access HBsAg assay demonstrated good analytical performance, equivalent recognition of seroconversion panels compared to Architect assay, and an analytical sensitivity between 0.022 and 0.025 IU/mL. All HBV genotypes, subtypes and mutants were well detected without analytical sensitivity loss.

**Conclusion:**

Access HBsAg and Access HBsAg Confirmatory assays demonstrated robust performances. They provide low samples volume requirements and a simplified process, no systematic retesting for high positive samples.

## Highlights

• Access HBsAg assays on DxI 9000 proved robust clinical and analytical performances.
• Low samples volume requirements.
• Simplified process; no repeating nor confirmatory testing for high positive samples.

## 1 Introduction

According to the World Health Organization (WHO), about 296 million people were living with chronic hepatitis B virus (HBV) infection in 2019. In the same year, HBV infections resulted in an estimated 820,000 deaths, mostly from cirrhosis and liver cancer. Despite the existence of vaccine and treatments for several decades, the number of infected people is on the rise, with 1.5 million new infections yearly [1], the highest burden occurring in the Western Pacific and African regions [2].

HBV transmission can occur through several routes ranging from sexual intercourse to horizontal/vertical transmission from mother/child to child [3]. After primary infection, the risk of chronic infection is dependent on the age and immune status of the host. Persistent HBV infection remains mostly asymptomatic despite extensive intrahepatic viral replication and may lead to cirrhosis, hepatic insufficiency, portal hypertension, and primary hepatocellular carcinoma [4,5].

As acute and chronic HBV infections are frequently asymptomatic, HBV antigen serological screening of at-risk groups on a regular basis is recommended by multiple worldwide guidelines [6,7]. In some countries, based on epidemiological and financial considerations, universal screening involving HBV testing at least once in adults aged ≥18 years is recommended [6,7].

HBV infection diagnosis is mainly based on serological assays. The hepatitis B surface antigen (HBsAg) is detected in high levels in serum during acute or chronic hepatitis B virus infection. It is the first serological marker to appear during primary infection, is used for definition of chronic hepatitis, and indicates that the person is infectious. Thus, initial HBV screening conducted with single HBsAg detection is currently the gold standard for active HBV infection diagnosis [5]. The WHO recommends confirming positive HBsAg with a neutralization step for settings with low HBsAg seroprevalence. Following a positive HBsAg serological test result, an HBV DNA nucleic acid test is recommended to guide treatment decisions in the absence of cirrhosis and enable monitoring [7]. A range of other HBV markers, such as anti-HBc Total and anti-HBc IgM, HBe antigen and antibodies, anti-HBs antibodies and HBV DNA can be used to monitor chronic HBV infection progression and response to treatment [5,7,8]. The “triple test combination” comprising HBsAg, anti-HBc IgG and anti-HBs IgG for initial screening appears to be cost-effective in some settings [8,9].

Standardized serological assays for HBsAg measurement utilize highly sensitive chemiluminescent microparticle immunoassay technology and their coupling with automated immunoassay systems have led to even improved sensitivity, specificity, and accuracy [10]. Nonetheless, there is the ongoing need for the development of even more efficacious methods capable of large-scale testing that provide faster turnaround times.

The objective of this study was to evaluate the performances of the new Beckman Coulter Access HBsAg and Access HBsAg Confirmatory assays on the DxI 9000 Access Immunoassay Analyzer.

## 2 Material and methods

### 2.1 Study design and participants

The study is a multicenter, prospective study using both prospectively and retrospectively collected patient samples (NCT04904835). Samples were residual fresh or thawed EDTA plasma or serum, with sufficient volume available to perform all required testing. They were collected from eligible subjects ≥18 years of age belonging to the following included groups: unselected blood donors, hospitalized patients and presumed HBsAg positive patients. Of a total of 7829 samples collected for the study, 295 were excluded. Among the 7534 samples included in the final analysis, most were serum (7241/7534) and the remaining were EDTA plasma. A portion (200/1032) of hospitalized patient samples and the majority of HBsAg positive samples (420/455) were collected and stored frozen prior to testing. Most hospitalized patient samples (832/1032), a portion of HBsAg positive patient samples (35/455) and all blood donor samples (6,047) were tested fresh. All hospitalized patient and blood donor samples and a portion of presumed HBsAg positive (105/455) were prospectively collected. When tested for other HBV serological markers with CE-marked assays, 12 presumed HBsAg positive subjects (2.6%) were classified as at the acute stage of the disease (positive for HBsAg, anti-HBc IgM and anti-HBc Total), whereas 442 (97.1%) were classified as chronic patients (positive for both HBsAg and anti-HBc Total, but negative for anti-HBc IgM). The last sample could not be tested for other HBV serological markers due to insufficient volume, but this sample came from a patient at the chronic stage of HBV infection based on the patient's medical record. Demographic data from the 7534 included specimens showed that 3592 were from males (47.7%) and median age ±SD was 45 years ± 16.6 years. All patients were from a European population.

Specificity was evaluated on blood donors and hospitalized patients' populations, while sensitivity of both Access HBsAg assay (herein, Access assay) and Access HBsAg Confirmatory assay (herein, Access Confirmatory assay) was evaluated on presumed HBsAg positive samples. Diagnostic sensitivity and specificity of Access and Access Confirmatory assays were evaluated by comparing their results to the HBsAg sample status determined in parallel by the CE marked HBsAg qualitative and HBsAg confirmatory assays (herein, Reference assays): Abbott Architect HBsAg Qualitative II assay (herein, Architect assay) and Abbott Architect HBsAg Qualitative II Confirmatory assay (herein, Architect Confirmatory assay) for presumed HBsAg positive and hospitalized patients, or Abbott PRISM HBsAg (herein, PRISM assay) and Abbott PRISM HBsAg Confirmatory assays (herein, PRISM Confirmatory assay), or Roche Elecsys HBsAg II (herein, Elecsys assay) and Architect Confirmatory assays for unselected blood donors. Anti-HBc total antibodies and IgM testing were requested, if not available at enrollment, for all presumed HBsAg positive patients’ samples for further characterization and serological classification of HBV infection, using CE marked anti-HBc IgM assays (Roche Elecsys Anti-HBc IgM or Abbott Architect Anti-HBc IgM assay) and anti-HBc total antibody assays (Roche Elecsys Anti-HBc II or Abbott Architect Anti-HBc II assay). These results were not used to determine final HBsAg sample status.

The study was performed from January 2020 to December 2021 in France at five (5) collection sites (Cerba Xpert, Saint Ouen L'Aumône; Eurofins Biomnis, Ivry-Sur-Seine; Etablissement Français du Sang (EFS), Bassins Normandie Est et Normandie Ouest, Bois Guillaume; Universitary hospital of Rouen, Rouen; Universitary hospital of Amiens, Amiens) and three (3) testing sites (Cerba Xpert, Saint Ouen L'Aumône; Eurofins Biomnis, Ivry-Sur-Seine; Etablissement Français du Sang (EFS), Bois Guillaume) on DxI 9000 Access Immunoassay Analyzers (herein, DxI 9000 system). Due to low prevalence of acute/recent HBV infected individuals, presumed HBsAg positive samples from patients at the acute stage of infection were purchased from a biospecimen provider (Biomex GmbH, Heidelgerg, Germany). All remnant samples collected or purchased were anonymized or pseudonymized (with documented oral patient consent). The study complied with the ethical principles of the Declaration of Helsinki and the International Conference for Harmonization Guidelines for Good Clinical Practices.

### 2.2 Test assays

The characteristics of the Access and Access Confirmatory assays are summarized in Table 1. The Access assay is a qualitative test using an assay signal to cutoff (S/CO). Results are reported to be “reactive” or “nonreactive” as a function of their relationship with the “cutoff” (signal “≥” or “<” 1.00 S/CO). Samples with S/CO values < 1.00 indicate absence of HBsAg (nonreactive, NR), while samples with values of S/CO ≥ 1.00 to <100.00 indicate presence of HBsAg (initial reactive, IR) which require to be repeated in duplicate. If both results of the duplicate were <1.00 S/CO, the sample is considered NR, otherwise the sample is considered as repeatedly reactive (RR) for HBsAg. All RR results require samples to be confirmed using the Access Confirmatory assay. Samples presenting S/CO ≥ 100.00 at initial testing are considered as high positive samples. There is no gray-zone or final equivocal result for the Access assay.

**Table 1:** Summary of test assay characteristics.

Assay name	Immunoassay System	Assay Type	Sample Input	Sample Type	Time to First Result	Testing Rate	Sample Result Unit of Measure
Access HBsAg assay	DxI 9000 Access Immunoassay System	One-step enzyme immunoassay	45 μL^a^	Serum (gel/no gel), Plasma^b^	29 min	450 tests/hr	Signal/Cutoff (S/CO)
							No gray zone
							High positive algorithm^c^
Access HBsAg Confirmatory assay	DxI 9000 Access Immunoassay System	Neutralization assay	90 μL	Serum (gel/no gel), Plasma^b^	29 min	450 tests/hr	Signal/Cutoff (S/CO)
							And % Neutralization
							No gray zone

a Does not include dead volume which varies based on sample container used.

b Lithium Heparin, Lithium Heparin gel, K2 EDTA, K3 EDTA, Sodium Citrate, Acid Citrate Dextrose (ACD), CPD.

c S/CO < 1.00 are negative, S/CO ≥ 100.00 are positive, S/CO ≥ 1.00 and < 100.00 require retest in duplicate for interpretation and samples with RR results should be confirmed using the Access HBsAg Confirmatory assay per manufacturer.

The Access Confirmatory assay uses the principle of neutralization by an excess of HBsAg-specific antibodies (neutralization reagent) to confirm the presence of HBsAg. Final Access Confirmatory assay interpretation is either confirmed, if the S/CO is ≥ 1.00 and percent neutralization is ≥ 40%, or NR, if the S/CO is < 1.00. Up to two sample dilutions at 1:250 and 1:62,500 may be required to get the final sample status.

### 2.3 Other performance testing

Detailed procedures for imprecision, sample carryover, cross-reactant samples, seroconversion panels, analytical sensitivity, sample type potential interference and recognition of HBV variants are provided in the supplemental methods.

Briefly, imprecision was assessed using four serum and four plasma samples at one internal site over 20–23 days with three Access and Access Confirmatory lots. Sample carryover contamination was assessed on two reagent packs using 5 cycles of a HBsAg-negative sample running subsequently to a sample with high HBsAg titer (0.5 mg/mL). The S/CO of the baseline after carryover and the S/CO of each negative sample after high HBsAg positive sample were compared with the S/CO of the baseline before carryover. 406 cross-reactant samples were tested using the Access assay. Sensitivity and specificity of Access HBsAg assay on cross reactant samples were evaluated by comparing results to the HBsAg sample status determined by the Reference assays. The sensitivity of the Access assay during the early phase of infection was evaluated using 30 seroconversion panel samples. HBV genotypes, subtypes and mutants detection of Access and Access Confirmatory assays were assessed by testing a panel of 24 samples containing genotypes A through H, 9 HBsAg subtypes and a total of 30 (10 native and 20 recombinant) HBsAg mutant samples. Finally, analytical sensitivity of Access assay was determined by testing dilution series of the WHO Third International Standard for HBsAg spiked.

### 2.4 Statistical analysis

Statistical analysis followed appropriate guidelines, (i.e., CLSI EP05-A3 [11], CLSI EP10-A3-AMD [12], CLSI EP07-ED3 [13] and CLSI EP17-A2 [14]) for imprecision, sample carryover, cross reactant and analytical sensitivity analyses, respectively. Evaluation on seroconversion panels, recognition of genotypes, subtypes and mutants, specificity and sensitivity analysis followed the CLSI EP12-A2 guidelines [15]. Diagnostic specificity and sensitivity of the Access and Access Confirmatory assays were evaluated by comparing the diagnostic classification of the Access assays to the final HBsAg sample status determined by the sample status algorithm. To validate the high positive algorithm for the Access HBsAg assay, the confirmed sensitivity was calculated on presumed HBsAg positive samples confirmed positive by sample status algorithm using the first result only for high positive samples, without considering the retest in duplicate, nor confirmatory test results. For non-high positive samples (samples with S/CO < 100.00), retest in duplicate as well as Access Confirmatory test results (if RR) were considered for the analysis. Point estimates in percentage, event counts, and two-sided 95% exact confidence intervals were calculated. Observed frequencies in NR, IR, RR and confirmed, and RR and not confirmed Access and Access Confirmatory assay results versus HBsAg status of sub-populations of interest were tabulated. Demographic data were reported in descriptive statistics. All statistical analyses were performed using SAS v9.4 (SAS Institute, Cary, NC, USA), R v4.1.3 (R Foundation for Statistical Computing, Vienna, Austria) and Microsoft Excel (Microsoft Corp. Redmond, WA, USA).

## 3 Results

### 3.1 Specificity analysis

**Fig. 1:**
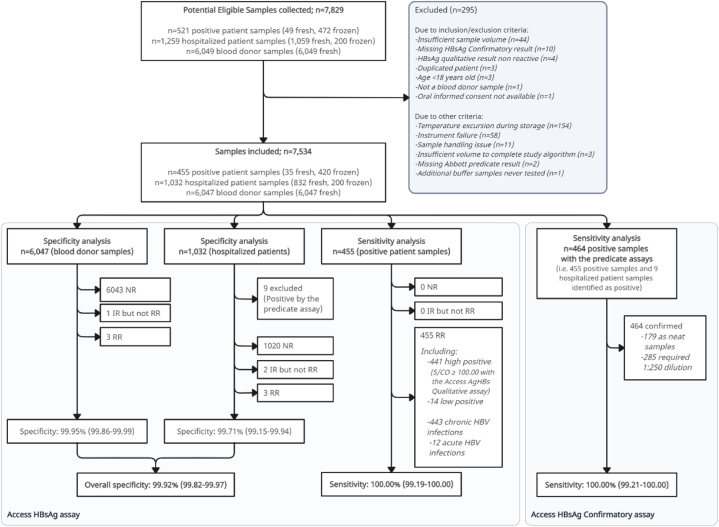
Flow chart of the study.

Fig. 1 summarizes the main results of the clinical study. Among the blood donor samples, 6044/6047 were NR, including one IR confirmed NR by the repeated duplicate test and three RR using the Access assay but not confirmed by the Access Confirmatory assay (Table 2). Access assay RR specificity on 6047 unselected blood donors was 99.95% (95% CI: 99.86%–99.99%). Out of 6047 blood donor samples tested during the clinical trial, 4299 were tested with both Access and Elecsys assays, 1747 were tested with both Access and PRISM assays, and one was tested with Access, Elecsys and PRISM assays. Overall %agreement of the Access assay vs. Elecsys assay was 99.93% (95% CI: 99.80%–99.99%). Overall %agreement of the Access assay vs. PRISM assay was 99.94% (95% CI: 99.68%–100.0%).

**Table 2:** Summary of results for non-selected blood donor, hospitalized patient and presumed HBsAg positive patient samples.

Group	N (%) of samples	Access HBsAg assay result	HBsAg Qualitative Reference assay result a	Access HBsAg Confirmatory assay result	HBsAg Confirmatory Reference assay result b	Final HBsAg Sample Status	Final Access HBsAg Sample status
Fresh Blood Donors	6043 (99.93)	NR	NR	NA	NA	NEG^c^	Nonreactive
	1 (0.02)	NR	RR^d^	NA	NR	NEG^c^	Nonreactive
	3 (0.05)	RR	NR	NR	NA	NEG^c^	Nonreactive
Fresh Hospitalized Patients	821 (98.68)	NR^e^	NR	NA	NA	NEG^f^	Nonreactive
	8 (0.96)	RR	RR	Confirmed	Confirmed	POS^g^	Confirmed positive
	3 (0.36)	RR	NR	NR	NA	NEG^f^	Nonreactive
Frozen Hospitalized Patients	199 (99.50)	NR	NR	NA	NA	NEG^f^	Nonreactive
	1 (0.50)	RR	RR	Confirmed	Confirmed	POS^g^	Confirmed positive
Fresh Presumed HBsAg Positive	35 (100.00)	RR	RR	Confirmed	Confirmed	POS^h^	Confirmed positive
Frozen Presumed HBsAg Positive	420 (100.00)	RR	RR	Confirmed	Confirmed	POS^h^	Confirmed positive

a Abbott Architect HBsAg Qualitative II assay for hospitalized and presumed positive samples and Abbott PRISM HBsAg or Roche COBAS Elecsys HBsAg II assay for non-selected blood donors.

b Abbott Architect HBsAg Qualitative II Confirmatory assay for hospitalized and presumed positive samples and Abbott PRISM HBsAg Confirmatory or Abbott Architect HBsAg Qualitative II Confirmatory assay for non-selected blood donors.

c Included for specificity on blood donors and overall specificity.

d One (1) blood donor sample RR with Roche Cobas Elecsys HBsAg II assay was tested NR for the presence of HBsAg with Architect HBsAg Qualitative II assay, Abbott PRISM HBsAg assay and Roche Cobas Elecsys HBsAg Confirmatory assay.

e Two (2) fresh hospitalized patient samples IR with Access HBsAg assay had a final NR result after duplicate testing.

f Included for specificity on hospitalized patients and overall specificity.

g Included for overall sensitivity.

h Included for sensitivity based on HBsAg positive patients confirmed positive by testing algorithm and overall sensitivity.

Among the 1032 samples from the hospitalized patients’ group, nine were RR per the Access assay, confirmed reactive by the Access Confirmatory assay and with a final HBsAg status of positive per the Reference assays (Table 2). These nine true positive (TP) samples were discarded from the specificity analysis. A total of 1020 hospitalized patient samples were NR, including two IR (finally NR after the repeated duplicate test), and three RR not confirmed as positive for HBsAg by the Access Confirmatory assay. Access assay RR specificity on 1023 negative hospitalized patient samples was 99.71% (99.15%–99.94%).

Overall, the Access assay IR and RR specificity on the 7070 total samples with final HBsAg status negative were 99.87% (99.76%–99.94%) and 99.92% (99.82%–99.97%), respectively. Of note, all six false RR results (three each from blood donors and hospitalized patients) were not confirmed with the Access Confirmatory assay and were finally considered as non-reactive.

### 3.2 Sensitivity among HBsAg positive patients

Among the samples from the presumed HBsAg positive patients, 455/455 were IR and RR using the Access assay. All were confirmed positive for HBsAg by the Access Confirmatory assay and the Reference assays (Table 2). The Access assay IR and RR sensitivity was 100.00% (95% CI: 99.19 %–100.0%). Including the nine samples from the hospitalized patients group confirmed positive for HBsAg, the IR and RR sensitivity of the Access assay was 100.00% (95% CI: 99.21%–100.0%) on the 464 samples positive for HBsAg. 441 out of the 455 presumed HBsAg positive and all nine samples from the hospitalized patient's group were high positive (i.e., S/CO ≥ 100.00; 96.98% of all HBsAg positive specimens).

### 3.3 False initial reactive rate (IRR) on fresh samples

The false IRR was calculated for the 6047 blood donor and 832 hospitalized patient samples, of which, 14 were confirmed TP on both Access and Reference assays and were excluded from the false IRR calculation. Only 2/823 negative fresh hospitalized samples and 1/6044 negative fresh blood donations were IR but had a final NR result after duplicate testing with Access assay, leading to a false IRR on the fresh samples of 0.04% (95% CI: 0.01%–0.13%).

### 3.4 Access HBsAg confirmatory assay

Among the 470 specimens which were RR per the Access assay (455 presumed HBsAg positive samples, 12 hospitalized patients' samples, and three blood donors’ samples), 464 were confirmed for the presence of HBsAg with Access Confirmatory assay and with Reference assays. Six samples (three each from blood donors and hospitalized patients) were finally NR for the presence of HBsAg with Access Confirmatory and Architect Confirmatory assay.

### 3.5 Validation of the high positive algorithm

All samples with Access assay result S/CO ≥ 1.00 were retested in duplicate, including the samples with S/CO ≥ 100.00 (high positive), and then all RR samples were confirmed for the presence of HBsAg using the Access Confirmatory assay with the intent to validate the use of the high positive algorithm (i.e. no need to retest in duplicate samples that were IR with S/CO ≥ 100.00 nor to run Access Confirmatory assay).

Among the 450 high positive specimens per the Access assay (441 from the presumed HBsAg positive and 9 from the hospitalized patient samples), all were finally TP. Thus, the sensitivity among the high positive samples was 100.00% (95% CI 99.19%–100.0%), validating the use of the high positive algorithm with the Access assay.

### 3.6 Confirmed specificity and sensitivity

Considering the whole HBsAg testing process, 6 out of 7070 samples with final HBsAg status negative (6047 blood donors and 1023 hospitalized patients) were false positive (FP) with the Access assay. None were confirmed for the presence of HBsAg using the Access Confirmatory assay and considered negative per manufacturer recommendations (overall specificity = 100.00% [99.95%–100.0]) for the Access Confirmatory assay.

Among the 455 confirmed HBsAg positive samples, all were positive with the Access assays. Thus, confirmed sensitivity of the Access Confirmatory assay was 100.00% (99.19%–100.0%). Considering the 464 total HBsAg positive samples and 9 confirmed positive samples from the hospitalized patients, the overall sensitivity was 100.00% (99.21%–100.0%).

### 3.7 Precision study

This evaluation expected a total of 360 measurements of each serum and plasma sample for the Access assay. Results showed a maximum repeatability (within-run) of 1.6% CV for positive patient samples and 3.2% CV (0.015 S/CO SD) for negative patient samples. The maximum patient sample reproducibility obtained was 4.5% CV on positive samples and 0.040 S/CO SD on negative samples (Table 3).

**Table 3:** Imprecision of Access HBsAg and Access Confirmatory assays.

Assays	Sample	N	Mean (S/CO)	Mean (% NT)	Repeatability (Within-Run)		Between-Run		Between-Day		Reproducibility	
					SD (S/CO)	%CV	SD (S/CO)	%CV	SD (S/CO)	%CV	SD (S/CO)	%CV
Access HBsAg	Serum Low neg	360	0.11	N/A	0.003	3.2%	0.001	1.1%	0.001	1.2%	0.005	4.3%
	Serum High neg	360	0.83	N/A	0.011	1.3%	0.026	3.1%	0.000	0.0%	0.040	4.9%
	Serum Low pos	360	1.09	N/A	0.017	1.6%	0.025	2.3%	0.000	0.0%	0.049	4.5%
	Serum High pos	360	217.91	N/A	3.019	1.4%	4.765	2.2%	0.000	0.0%	9.377	4.3%
	Plasma Low neg	360	0.10	N/A	0.003	2.7%	0.002	2.1%	0.001	0.5%	0.005	4.7%
	Plasma High neg	360	0.86	N/A	0.015	1.7%	0.015	1.7%	0.006	0.6%	0.039	4.6%
	Plasma Low pos	360	1.01	N/A	0.015	1.5%	0.023	2.3%	0.009	0.9%	0.046	4.5%
	Plasma High pos	360	222.43	N/A	3.467	1.6%	4.729	2.1%	0.000	0.0%	9.044	4.1%

### 3.8 Sample carryover

Five cycles of a high HBsAg positive specimen (0.5 mg/mL) and a nonreactive serum were tested in two runs using different reagent packs. For each of the two runs and for the two runs averaged together, there was no significant trend (P > 0.05) of the S/CO bias across the five cycles of carryover nor pack contamination the day after. The maximum S/CO bias just after high HBsAg positive sample was 0.13 S/CO while the difference between mean S/CO bias of the baseline before and after carryover had a maximum bias of 0.04 S/CO.

### 3.9 Specificity on cross-reacting samples

A total of 406 samples from 41 different categories of cross-reactants were tested on the Access and Architect assays (Table 4). Of these, 5 were found reactive and confirmed per the Access and Architect assays, corresponding to a sensitivity of 100.00% (95% CI: 99.93%–100.0%) for both assays. All 401 remaining cross reactant samples were NR per the Access assay while 8 samples were found IR with Architect assay but not confirmed with Architect Confirmatory assay.

**Table 4:** Summary of results for cross-reactant samples.

Category/Marker	Number of samples tested	Access HBsAg assays result		Architect HBsAg assays result	
		# non-reactive^a^	# initially reactive/confirmed reactive^b^	# non-reactive^a^	# initially reactive/confirmed reactive^b^
**Infectious and related diseases**					
Epstein-Bar virus (EBV)/anti-EBNA IgG	10	10	0/0	10	0/0
Cytomegalovirus (CMV)/anti-CMV IgG and IgM	10	10	0/0	8	2/0
Herpes Simplex virus (HSV1/2)/anti-HSV1/2 IgG	10	10	0/0	10	0/0
Human Immunodeficiency Virus (HIV)/p24, anti-HIV-1/2 IgG	10	10	0/0	10	0/0
Hepatitis A Virus (HAV)/anti-HAV IgG and IgM, RNA, DNA	10	10	0/0	10	0/0
Hepatitis C Virus (HCV)/anti-HCV IgG and IgM	10	10	0/0	10	0/0
Hepatitis E Virus (HEV)/anti-HEV IgG	10	10	0/0	9	1/0
Alcoholic liver disease/Alanine Transaminase (ALT) and Aspartate Transaminase (AST)	10	10	0/0	10	0/0
Primary biliary cirrhosis/ALT and AST	10	10	0/0	10	0/0
Flavivirus (Co-infected Dengue and Zika)/anti-Zika IgG and anti-Dengue IgG	2	2	0/0	1	1/0
Flavivirus (Zika)/anti-Zika IgG and IgM	11	11	0/0	10	1/0
Flavivirus (Dengue)/anti-Dengue IgG	12	8	4/4	8	4/4
Toxoplasmosis/anti-Toxoplasmosis IgG	10	10	0/0	9	1/0
Syphilis/anti-TP IgG and IgM	10	10	0/0	10	0/0
Flavivirus (West Nile)/anti-West-Nile IgG, RNA	10	10	0/0	10	0/0
***Microbial interferences***					
*S. aureus*	10	10	0/0	10	0/0
*P.aeruginosa*	10	10	0/0	10	0/0
*E.coli*	10	10	0/0	10	0/0
***Vaccination related interferences and auto-immune diseasesv***					
Influenza Post Vaccination	10	10	0/0	10	0/0
Human Anti-Mouse Antibody (HAMA)	10	10	0/0	10	0/0
Anti-nuclear Antibody (ANA)	10	10	0/0	10	0/0
Rheumatoid Factor (RF)	10	10	0/0	10	0/0
Systemic lupus erythematosus (SLE)/Sm/RNP positive	10	10	0/0	10	0/0
***Pregnant related interferences***					
Pregnancy (multipara)	10	10	0/0	10	0/0
Pregnancy (first trimester)	10	10	0/0	10	0/0
Pregnancy (second trimester)	10	10	0/0	10	0/0
Pregnancy (third trimester)	10	10	0/0	10	0/0
***Antigen interferences***					
Cytomegalovirus (CMV) antigen	10	10	0/0	10	0/0
Epstein-Bar virus (EBV) antigen	10	10	0/0	10	0/0
Hepatitis A Virus (HAV) antigen	10	10	0/0	10	0/0
Hepatitis C Virus (HCV) antigen/Core, NS3, NS4	10	10	0/0	10	0/0
Hepatitis E Virus (HEV) antigen/ORF2 and ORF3	10	10	0/0	10	0/0
Human Immunodeficiency Virus (HIV) antigen/HIV-1 p24	10	10	0/0	10	0/0
Herpes Simplex virus (HSV2) antigen	10	10	0/0	10	0/0
Rubella antigen	10	10	0/0	10	0/0
Varicella Zoster Virus (VZV) antigen/ORF9	10	10	0/0	10	0/0
Toxoplasmosis antigen	10	10	0/0	10	0/0
Syphilis antigen/*Treponema pallidum* p15, p17, p47	10	10	0/0	10	0/0
***Others***					
Transplant, Transplant recipient	10	10	0/0	9	1/0
Dialysis patients	11	10	1/1	9	2/1
Hemophiliac, Clotting factor/ADAMTS13 antibody	10	10	0/0	10	0/0
**Total**	**406**	**401**	**5/5**	**393**	**13/5**

a For both Access HBsAg and Abbott Architect HBsAg assays, cross-reactant specimens were counted as “non-reactive” if first result was <1.00 S/CO.

b If first result was ≥1.00 S/CO, samples were counted as “initially reactive” and tested directly in Access HBsAg Confirmatory or Abbott Architect HBsAg Confirmatory assay. All “initially reactive” cross-reactant specimens were also counted as “confirmed reactive” if HBsAg positivity was confirmed in Access HBsAg Confirmatory or Abbott Architect HBsAg Confirmatory assay.

### 3.10 Seroconversion panels

Thirty HBsAg commercial seroconversion panels, including a total of 336 bleeds, were tested with the Access and Architect assays. The Access assay performed later for three panels but equivalent on 27 panels in terms of detection day of first positive bleed compared to the Reference assay. The difference of mean first day of detection across the 30 seroconversion panels was less than one day (0.7 days) between the two assays.

### 3.11 Recognition of HBV mutants, genotypes, and subtypes

All 30 mutant specimens diluted at concentrations close to cutoff (1.00–6.27 S/CO) on the Architect assay were detected by the Access assay with S/CO between 1.05 and 3.23 S/CO and confirmed with the Access Confirmatory assay. All 24 samples containing genotypes A through H and the 9 commercially available subtypes were also detected and confirmed by Access assays.

### 3.12 Analytical sensitivity

**Fig. 2:**
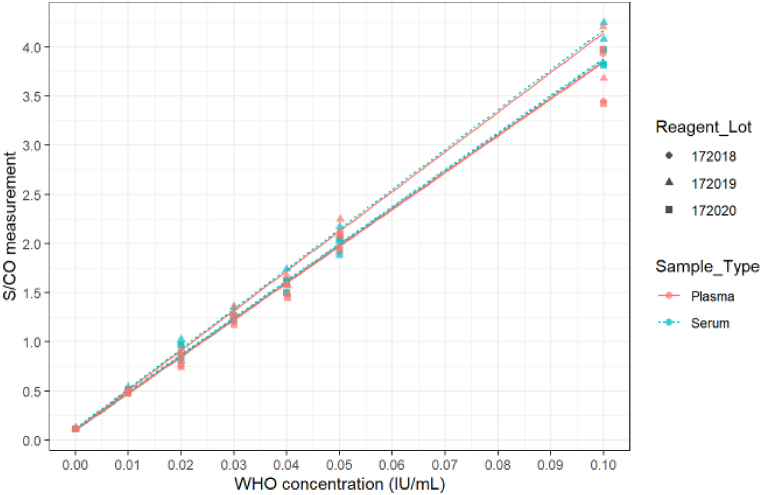
WHO 3rd International Standard HBsAg analytical sensitivity of the Access HBsAg assay.

The analytical sensitivity of Access assay on the WHO's Third International Standard was found to be between 0.022 IU/mL (95% CI: 0.022–0.022 IU/mL) and 0.025 IU/mL (95% CI: 0.024–0.026 IU/mL) across reagent lots and sample types (Fig. 2).

### 3.13 Sample type

A sample type study was conducted to assess any potential interference which would occur in the different common anticoagulants in test tubes (serum, serum separator tube, lithium heparin, lithium heparin separator tube, dipotassium EDTA, tripotassium EDTA, sodium citrate, acid citrate dextrose and citrate phosphate dextrose). A total of 40 negative and 40 positive panels, spiked at different HBsAg concentrations and containing the 9 tube types each, were tested in duplicates on one reagent lot of Access assay. Maximum average bias concentration on the 40 negative panels was obtained using tri-potassium (K3) EDTA tubes with a 0.022 S/CO bias compared to the reference serum sample type. As well, maximum average % bias concentration obtained from testing of the 40 positive panels was using lithium heparin tubes with a −2.8% bias compared to the reference serum sample type (see Table 5).

**Table 5:** Summary of results for sample types.

Sample Type	Average bias concentration (S/CO) vs. Reference (Serum No Gel) on Negative samples (<1.00 S/CO)	Average % bias concentration (%) vs. Reference (Serum No Gel) on Positive samples (≥1.00 S/CO)
Serum separator tube	0.000	−0.8%
Lithium Heparin	0.000	−2.8%
Lithium Heparin separator tube	0.000	−1.9%
Dipotassium (K2) EDTA	0.007	1.0%
Tripotassium (K3) EDTA	0.022	1.5%
Sodium Citrate	0.003	0.0%
Acid Citrate Dextrose (ACD)	0.003	−1.2%
Citrate Phosphate Dextrose (CPD)	0.003	−0.7%

Access assay demonstrates consistent signals in the 9 main sample types with maximum bias within the imprecision of the assay compared to the reference serum tubes for both negative and positive samples. Access assay can be used indifferently whatever the sample type used in clinical tests.

## 4 Discussion

HBV is a highly infectious disease with an increasing prevalence worldwide despite vaccine and treatment availability. It represents the main cause of chronic hepatitis, cirrhosis, and hepatocellular carcinoma worldwide. Large serological diagnosis is a cornerstone of HBV infection fight and continuous efforts are made to develop HBsAg assays that are highly reliable, sensitive, provide high throughput and based on easier algorithms.

Combined with the enhanced chemiluminescent substrate of the DxI 9000 system [16], the new Access assay has analytical sensitivity on the WHO 3rd International Standard between 0.022 and 0.025 IU/mL. Access and Access Confirmatory assays were able to detect and confirm all tested HBV genotypes and subtypes as well as a panel of 30 mutants among the most frequently found. Escape mutants are a concern as they are the major cause of HBV reactivation, reinfection or occult infections in infected orthotopic liver transplantations recipients [17]. The Access assay also demonstrated comparable performance versus the Architect assay in detecting early HBV acute phase as the difference of mean first day of detection across the 30 seroconversion panels was 0.7 days between the two assays.

On a large cohort of 7534 samples, the clinical sensitivity of the Access assay observed in the current work among 464 HBsAg positive samples was 100.00% (99.21%–100.0%). It is comparable to those reported by other major HBsAg assays such as Architect assay, HBsAg Next Qualitative on Architect and on Alinity i, Elecsys and LIAISON XL HBsAg assays with reported clinical sensitivities of 99.71% (98.39–99.99), 100.00% (99.18%–100.0%), 100.00% (99.17%–100.0%), 99.90% (99.37%–99.99%) and 100.00% (99.20%–100.0%) respectively [[18], [19], [20], [21]].

Specificity of the Access assay for blood donors (99.95% [99.86%–99.99%]), and hospitalized patients (99.71% [99.15%–99.94%]) met the standards of other high throughput assays. Indeed, in its package insert, the Elecsys assay claims a clinical specificity at 99.98% and 99.88% on blood donors and daily routine samples including hospitalized patients, respectively [20]. The LIAISON XL HBsAg assay claims clinical specificities of 99.93% and 99.75% on blood donors and hospitalized patients, respectively. The clinical specificities on blood donors of the Architect assay, the HBsAg Next Qualitative assay on Architect and on Alinity i have been reported at 99.92% (99.82%–99.98%), 99.95% (99.87%–99.99%) and 99.96% (99.87%–99.99%), respectively. Overall, one false IR out of 6044 fresh NR blood donors and two false IR out of 821 fresh NR hospitalized patients were found with the Access assay leading to an overall false IRR of 0.04% (3/6865). This assessment remains a worst-case as only fresh samples were considered to estimate the false IRR. It was in line with reference assays on the market as in their package inserts, the overall false IRR of LIAISON XL MUREX HBsAg and Architect assays across blood donors and hospitalized patients were 0.10% (6/5791) and 0.03% (2/6383) respectively.

The Access assay has reproducibility CV% in the range 4.1–4.5% for positive samples and reproducibility SD in the range 0.005–0.040 S/CO for negative samples. As a comparison, the LIAISON XL HBsAg assay claims a reproducibility CV% in the range 3.9%–6.3% for positive samples and reproducibility SD in the range 0.044–0.069 S/CO for negative samples [21]. Within-laboratory precision (Total) of Architect assay was 2.1%–5.1% (CV%) for positive samples and 0.031–0.061 S/CO (SD) for negative samples [22]. Further confirmation of the accuracy of the Access assay is demonstrated by estimation maximum C5 (concentration below which <5% of the results are positive) and C95 (concentration above which >95% results are positive) of 0.94 S/CO (0.91–0.98 S/CO) and 1.07 S/CO (1.03–1.11 S/CO).

Besides the good analytical and clinical performance, reliable and straightforward algorithms, avoiding unnecessary repeated and confirmatory testing, is an important challenge. This allows inexpensive and more quickly available results without any additional delay for clinician and patients’ management. Optimization of the formulation of Access assay combined to high reliability of the DxI 9000 system, lead to highly reduced non-specific background, cross contaminations, and noise levels. Most of the cross-contaminations, a major concern for all HBsAg assays, usually occur during sample handling steps either by the operator or in the analyzer. FP results also lead to unnecessary repeat and confirmation testing, delaying the release of results and adding costs. In the new DxI 9000 system, dedicated the sample precision pipettor uses disposable tips to deliver samples to all four reagent build stations [23]. No negative sample contamination was detected when using the Access assay even after an extremely high HBsAg concentration sample at 0.5 mg/mL.

Finally, the high positive algorithm, (i.e., the possibility of interpreting as positive samples with first result ≥100.00 S/CO) using the Access assay without additional duplicate test nor confirmation, is validated in the current work without any FP observed among the 450 high-positive samples. Such algorithm implementation will permit the reduction of the burden of testing on a high portion of positive samples, 96.98% of the total HBsAg positive patient samples in the current work. To date, such an approach has only been proposed by the ADVIA Centaur HBsAg II assay.

This study did have some limitations. The study was a cross-sectional testing study, with no additional clinical information or follow-up available on patients. Even if this study is multicentric and with precautions to maximize testing conditions variability, the real-life performances can be slightly lower than those seen during initial evaluation done in a controlled manner. Independent assessment could be useful to confirm the strong performances observed in the current work.

In conclusion, the newly developed Access and Access Confirmatory assays, for use on the DxI 9000 system demonstrated similar specificity and sensitivity performances than currently marketed qualitative HBsAg high throughput commercialized assays. It also provides interesting features regarding its low sample volume requirements, robustness, and simplified processes that do not require systematic retesting nor assay confirmation for high positive samples.

## Research funding

This study was supported and funded by Beckman Coulter, Inc.

## Informed consent

For all samples that were fully anonymized leftover samples with no possibility to trace back to patient identity, no written informed consent was required per EU General Data Protection Regulation (GDPR). For all samples that were pseudonymized leftover samples, an oral informed consent was requested and documented in the eCRF.

## Ethical approval

Research involving human subjects complied with all relevant national regulations, institutional policies and is in accordance with the tenets of the Helsinki Declaration (as revised in 2013). Research using anonymized, leftover specimens does not require specific ethical approval in France as it falls outside of the Loi Jardé (n°2012-300 of March 5, 2012; amended by ordinance n°2016-800 of June 16, 2016).

The DxI 9000 ACCESS Immunoassay Analyzer is CE marked and FDA cleared but not currently for sale or distribution in all markets.

MAPSS Number 2023-12288.

## CRediT authorship contribution statement

**Benoit Visseaux:** Writing – original draft. **Jérémie Gautier:** Supervision, Investigation. **Françoise Le Boulaire:** Supervision, Investigation. **Catherine Coignard:** Supervision, Investigation. **Claire Vincent:** Supervision, Investigation. **Sandrine Gréaume:** Supervision, Investigation. **Isabelle Voisin:** Supervision, Investigation. **Veronique Lemée:** Supervision, Investigation. **Jean-Christophe Plantier:** Supervision, Investigation. **Yves-Edouard Herpe:** Supervision, Investigation. **Etienne Brochot:** Investigation. **Stephanie Bord:** Supervision, Methodology, Conceptualization. **Marc Turini:** Supervision, Methodology, Conceptualization. **Vanessa Roulet:** Supervision, Methodology, Conceptualization. **Juliane Hey:** Validation, Supervision, Methodology, Conceptualization.

## Declaration of competing interest

B.V., J.G., F.B., C.C., C.V., S.G., I.V., V.L., J-C.P., Y-E.H., E.B. declare no conflicts of interest, none of them received personal fees from Beckman Coulter and all are employees of institutions or companies paid by Beckman Coulter to perform this study. V.R., J.H., S.B. and M.T. are employees of Beckman Coulter.

## Data Availability

Data will be made available on request.
