# Supplementary material for: Performance evaluation of the Access HBsAg and Access HBsAg confirmatory assays on the DxI 9000 Access Immunoassay Analyzer
Source: Pract Lab Med. 2024 Mar 15;39:e00390. doi: 10.1016/j.plabm.2024.e00390 (PMC11075052; doi:10.1016/j.plabm.2024.e00390)
Supplement: Multimedia component 1 [file mmc1.docx]

**Supplemental Methods**

1. **Imprecision**

Two studies were performed at one internal site over 20-23 days with three Access HBsAg assay lots and three Access HBsAg Confirmatory assay lots. Four serum and four plasma samples, including contrived samples were used for the imprecision assessment. The sample panel included low and high negative samples and low and high positive samples. Samples were tested as 2 runs/day and 3 replicates/run.

1. **Sample Carryover**

Sample carryover contamination was assessed using an HBsAg-negative sample running subsequently to a sample with high HBsAg titer. Briefly, a negative f serum spiked with human HBsAg antigen (named high-positive sample; HBsAg at approximately 0.5 mg/mL) and the same negative sample without HBsAg were used. Two separate runs, done on two different reagent packs, were conducted. In each run, 5 replicates of negative sample (baseline before carryover) were run immediately followed by 5 cycles of the high-positive sample and negative sample. The day after, 5 replicates of negative sample (baseline after carryover) were finally run to assess potential pack contamination. The S/CO of the baseline after carryover and the S/CO of each negative sample after high HBsAg positive sample were compared with the S/CO of the baseline before carryover.

1. **Cross-reactant Samples**

A total of 406 samples from 41 different cross-reactant categories were tested in duplicate using the Access HBsAg assay. All samples were collected from commercial vendors and tested across three (3) lots of Access HBsAg assay. For preparation of bacterial and antigen specimens, negative samples were spiked with bacteria or antigen prior to evaluation. Sensitivity and specificity of Access HBsAg assay on cross reactant samples were evaluated by comparing results to the HBsAg sample status determined in parallel by the Architect HBsAg Qualitative II and Architect HBsAg Qualitative II Confirmatory assays.

1. **Seroconversion Panels**

30 seroconversion panel samples (Zeptometrix /Biomex) representing a total of 336 bleeds were used to evaluate the sensitivity of the Access HBsAg and Access HBsAg Confirmatory assays during the early phase of infection. Panels were tested in parallel with the Architect HBsAg Qualitative II and Architect HBsAg Qualitative II Confirmatory assays

1. **Recognition of HBV Genotypes, Subtypes, and Mutants**

HBsAg detection was assessed by testing a panel of 24 samples containing genotypes A through H (A (4), B (3), C (4), D (4), E (2), F (3), G (1) and H (3)), 9 commercially available HBsAg subtypes (adw2 (1), adw4 (1), adr (1), ayw1 (1), ayw2 (1), ayw3 (2), ayw4 (1) and ayr (1)) and a total of 30 (10 native and 20 recombinant) HBsAg mutant samples, containing defined mutations between amino acid 100 and 170. These 30 mutants represent the most frequent immune-escape mutations on HBV: Q129R+G130N, T126N, G130R+S132Y, P120S+F134I, T126A, Q129H+D144A, T118A (2 samples), Y137F, M133L, P142S, S143L, G145R, C137W, P142S+G145R, 122NT, C124R, D144A, F134H, G130N, K122T, L109I, M133T, P127T, Y161F, Q129H, T123N, T126S, T131A and Y100C. The mutant samples were diluted close to cut off (1.00 – 6.27 S/CO) on Architect HBsAg assay and tested on the Access HBsAg and Access HBsAg Confirmatory assays.

1. **Analytical Sensitivity**

HBsAg analytical sensitivity of Access HBsAg assay was determined by testing dilution series of the WHO Third International Standard for HBsAg (HBV genotype B4, HBsAg subtypes ayw1/adw2, NIBSC code:12/226) spiked in HBsAg negative human serum or plasma. The dilutions were assayed using three reagent lots on two DxI 9000 Access Immunoassay Analyzers over three days. The analytical sensitivity for each reagent lot was determined using a linear fit regression of the S/CO versus the WHO concentrations (IU/mL).

1. **Sample Type**

Individual donors were collected in the following 9 sample types: Serum, Serum separator tube, Lithium Heparin, Lithium Heparin separator tube, Dipotassium (K2) EDTA, Tripotassium (K3) EDTA, Sodium Citrate, Acid Citrate Dextrose (ACD) and Citrate Phosphate Dextrose (CPD). 40 panels were used untouched (negative samples), 10 were spiked with HBsAg to reach signal between 1.00 and 5.00 S/CO (low positive samples), 10 were spiked to reach signal between 5.00 and 10.00 S/CO (mid positive samples), 10 were spiked to reach signal around 100.00 S/CO and 10 were spiked to reach signal around 500.00 S/CO. The 9 tube types from the 40 negative and 40 positive panels were tested in 2 replicates using one reagent pack lot of Access HBsAg assay on one instrument. For each tube type of the 40 negative panels, the average bias concentration (S/CO) was calculated compared to the Serum tube type considered as the reference sample type and the percentage bias concentration (%) was calculated compared to the Serum tubes for each tube type of the 40 positive panels.
